# Supplementary material for: Optimization of running-in surface morphology parameters based on the AutoML model
Source: PLoS One. 2021 Oct 4;16(10):e0257850. doi: 10.1371/journal.pone.0257850 (PMC8489728; doi:10.1371/journal.pone.0257850)
Supplement: Attachment — Submitted filename: Response to Reviewers.docx [file pone.0257850.s001.docx]

The authors wish to thank the Editors and the anonymous reviewers very much for their valuable comments and suggestions, which leads to significant improvement of the quality and presentation of the manuscript. In the following, we will explain how the reviewer' comments have been considered in the revision.

**Suggestions from Chief editor:**

1. **The authors need to do thorough proofreading of this paper. Some phrases don't make sense and there are lots of typos and wrong use of prepositions.**

We are extremely sorry for the typos and grammatical errors. The manuscript has been carefully checked by authors and revised by a professional English language editing company to improve the grammar and readability.

2. **The authors should emphasize the difference between other methods to clarify the position of this work further.**

We highly appreciate the editor for this comment. We have accordingly revised the introduction, discussion and conclusions in the revised manuscript. Through comparative analysis of literatures in relevant fields, we have clarified the positioning of this work. At the same time, we have added relevant content to clarify the rationality of the method selection.

**Reviewers' comments:**

Reviewer #1:

1. Authors should improve the overall readability of the paper.

**Author response:** The manuscript has been carefully checked by authors and revised by a professional English language editing company to improve readability.

2. There should a related work section showing the latest references and comparison of this study with existing ones.

**Author response:** We are extremely grateful for this comment. We have rewritten the content of the introduction part. At the same time, we have added and deleted some references in the revised manuscript. Through comparative analysis of literatures in relevant fields, we have clarified the positioning of this work.

3. There are many typos and grammatical mistakes in the entire paper.

**Author response:** We are extremely sorry for the typos and grammatical errors. The manuscript has been carefully checked and revised.

4. All the key terms of the equations must be mentioned

**Author response:** Thank you for your comment. We have included the relevant content in the revised manuscript.

5. The authors should further add explanation about research method.

**Author response:** Thank you for your valuable advice. We have added relevant content to clarify the rationality and correctness in 4.3. Method selection.

6. What are the evaluations used for the verification of results?

**Author response:** We appreciate the reviewer for the comment and have supplemented the relevant content (Section 5.3. Result verification) related to the verification of results in the revised manuscript. To verify the rationality of this model, three different working conditions were set in the running-in wear experiment. The prediction of model verification presents good prediction accuracy and shows good model generalization capability, and the results from model testing and verification are consistent.

7. Major contribution was not clearly mentioned in the conclusion part

**Author response:** Thank you for your comment. We have revised the relevant content in the conclusions of the revised manuscript. We have clarified the main contribution of the article in the revised manuscript. This work has provided a solution to establish linkages between design of unworn surface and performance of worn surface after running-in. Furthermore, the conclusions fill the gap in quality monitoring of lifecycle.

8. For ANN, Authors can cite the following paper.
   1) DeepAMD: Detection and identification of Android malware using high-efficient Deep Artificial Neural Network, Future Generation Computer Systems 115, 844-856
   - for bagging authors can cite below paper:
   A Novel PCA-Firefly Based XGBoost Classification Model for Intrusion Detection in Networks Using GPU

**Author response:** Thank you for your kind advice. We have supplemented these references in the revised manuscript.

Reviewer #2:

1. Introduction needs to explain the main contributions of the work more clearly.

**Author response:** Thank you for pointing this out. We have rewritten the content of the introduction section and added some content to explain the main contributions of this work more clearly.

2. Authors should improve on their language and grammar. I advise seeking help from a native English reader.

**Author response:** We are extremely sorry for the typos and grammatical errors. We have carefully checked this article and updated the manuscript by inviting a professional English language editing service to revise it carefully in order to avoid language errors and typos.

3. Literature review techniques have to be strengthened by including the issues in the current system and how the author proposes to overcome the same.

**Author response:** We appreciate the reviewer for this comment. We have updated the manuscript by rewriting the introduction. We have introduced the importance of running-in wear research and analyzed the present situation and limitations of related research. Furthermore, through the analysis and research of the relevant literatures in the relevant fields, we have proposed to apply machine learning methods to the field of running-in wear.

4. Hyperlinks are not working for the equations and figures

**Author response:** Thank you for your comment. We have amended the relevant parts in the revised manuscript.

5. The below article can be cited if needed
   Analysis of dimensionality reduction techniques on big data

**Author response:** Thank you for your kind advice. We have supplemented this reference in the revised manuscript.
